# The Relationships between Adolescents’ Obesity and the Built Environment: Are They City Dependent?

**DOI:** 10.3390/ijerph16091579

**Published:** 2019-05-06

**Authors:** Neta HaGani, Mika R. Moran, Or Caspi, Pnina Plaut, Ronit Endevelt, Orna Baron-Epel

**Affiliations:** 1 School of Public Health, University of Haifa, Haifa 3498838, Israel; moran.mika@gmail.com (M.R.M.); ronit.endevelt@moh.gov.il (R.E.); orna.epel@gmail.com (O.B.-E.); 2 Faculty of Architecture and Town Planning, Technion- Israeli Institute of Technology, Haifa 3200003, Israel; orcaspi@gmail.com (O.C.); pninatech@gmail.com (P.P.)

**Keywords:** walkability, adolescents’ obesity, built environment, physical activity, nutritional environment, sedentary behaviors

## Abstract

There is evidence that the built environment can promote unhealthy habits which may increase the risk for obesity among adolescents. However, the majority of evidence is from North America, Europe and Australia, and less is known about other world regions. The purpose of this study was to examine how the number of overweight and obese adolescents may vary in relation to the built environment, area socioeconomic status (SES), physical activity (PA) and nutritional home environment. We performed a telephone survey of 904 adolescents ages 15–18 from three different cities in Israel. The questionnaire included: reported PA, sedentary behaviors and nutritional home environment. Body Mass Index (BMI) was attained from records of Maccabi Healthcare Services (MHS). The built environment measures were calculated by Geographic Information System (GIS). Multivariable logistic regression analysis was performed to identify variables associated with adolescents’ overweight and obesity. The highest level of overweight and obese adolescents was in Beer Sheva (29.2%). The three cities did not differ in built environment characteristics, PA and sedentary behaviors. In Haifa, a more positive nutritional home environment was reported (*p* = 0.001). Boys, in all three cities presented higher rates of overweight and obesity (29%). After adjusting for covariates, adolescents’ overweight and obesity was associated with built environment measures only in a low SES peripheral city (OR = 0.72; 95% CI: 0.56–0.92), and positively associated with higher level of sedentary behavior in the total sample (OR = 1.23; 95% CI:1.03–1.47). This may imply a much more complex causal pathway between the built environment, SES and obesity than suggested in previous literature.

## 1. Introduction

### 1.1. Background

Adolescents’ obesity has become a worldwide epidemic [1] and was described by the World Health Organization (WHO) as one of the most serious public health challenges of the 21st century [2]. Obesity has been associated with unhealthy lifestyle, education, income and environmental factors [3,4]. Childhood and adolescence obesity has increased world-wide in the last 40 years with the number of children aged 5–19 years with obesity rising from 11 million in 1975 to 124 million in 2016 This increase is especially high in low-middle income countries. In high income countries, obesity rate remains very high even though it seems like it reached a plateau [5]. Similar trends are observed in Israel [6] showing that Israel is ninth (of 44 countries) in obesity rates among 15-year-old adolescents (14% girls and 24% boys) [7]. However, data from a national survey among 12–18 year old adolescents showed a decrease in obesity, with 14.2% during the years 2005–2007 and only 10% between 2010 and 2012 [8].

Poor diet and lack of physical activity (PA) are the main risk factors for childhood and adolescence obesity [9]. Only a fifth of adolescents worldwide are following the WHO guidance for at least 60 min of PA per day [10,11]. Sedentary behaviors involving screens (e.g., computer, television and phone) have also been associated with obesity and with numerous physical and mental health conditions across childhood and adolescence [12,13]. Children’s nutritional habits are formed in the home environment, which consists of the availability of certain foods in the home as well as family rules and limits regarding food intake [14]. The home environment has been associated previously with children’s consumption of fruits and vegetables and soft drinks, as well as with obesity rates [15,16]. Parental consumption of certain foods was also found to be crucial in explaining children’s nutritional behaviors like: fruit and vegetable intake, fat intake, breakfast and soft drink consumption [17,18].

There is evidence that the neighborhood built environment and the nutritional home environment can promote unhealthy eating and sedentary habits which may increase the risk for obesity [19]. A large body of literature has adopted the concept of walkability to describe the extent in which the built environment promotes walking and other forms of active transportation [20]. Walkable environments were found to be associated with healthier nutritional and PA behaviors [21,22]. However, most of the studies that found associations between the built environment and health behaviors were conducted in Western or developed countries like USA, Australia and Europe [23,24]. In many other non-Western countries like Latin America and East Asia, these relationships were found to be partial or non-existing [25,26]. A few studies from the Middle East found relationships between PA, nutrition and the built environment. However, these relationships were highly dependent on socioeconomic status (SES) of the child’s parents or neighborhood [27,28].

Evidence of the relationship between children and adolescents’ obesity and the built environment has been inconsistent across studies [29,30]. In some studies, lower Body Mass Indexes (BMIs) among children and adolescents were found to be associated with high proximity to green space and residential land uses [31,32,33]. However, other studies did not find associations between the built environment and obesity rates [3,34]. In many studies, SES mediated the relationship between the built environment attributes and adolescents’ BMI [3,35,36,37] and others found little effect of the built environment on adolescents’ BMI [22]. Similarly, in Israel, adolescents’ obesity was found to be more prevalent in cities characterized by lower socioeconomic rank and higher peripherality levels. Namely, wealthier cities that are located in the center of the country have lower risk of obesity. This is true only for the Jewish adolescent population in Israel and not the Arab adolescent population [38]. Differences in BMI, PA and nutrition have been previously found between adolescents from the Arab and Jewish populations in Israel. Jewish adolescents reported higher levels of PA and had lower rates of overweight and obesity compared to Arab adolescents. Arabs had also higher rates of eating sweets and fast foods compared to Jews [39,40].

The obesity epidemic among adolescents today will have a profound effect on the incidence of chronic diseases, such as diabetes, heart disease and cancer, in the future. Although there is evidence for the relationship between adolescence obesity and the built environment, it is not clear how much the built environment contributes to obesity compared to SES and other individual factors and if this is applicable to other countries and societies beyond those mostly researched to date (i.e., North America, Europe, Australia). It is also not clear which factors are the more important ones in relation to obesity among adolescents. 

### 1.2. Objectives

The overall objective of this study was to examine the associations between the built environment and overweight and obesity among Jewish adolescents aged 15–18 from three cities in Israel, while adjusting for area SES, PA, sedentary behaviors and nutritional home environment.

## 2. Materials and Methods 

### 2.1. Study Procedures and Participants

The sample recruited in this study includes 904 Jewish adolescents (ages 15–18) who participated in a telephone survey residing in three large cities in three different regions in Israel: north (Haifa), center (Rishon LeZion), south (Beer Sheva). Based on prior research associating adolescents BMI with city-level SES and peripherality [38], these cities were selected to represent a variety of socioeconomic levels, levels of peripherality and natural conditions. Haifa, the third largest Israeli city (279.6 thousand inhabitants), is a northern city situated on the Carmel mountain and by the sea, 77.5 km north of Tel Aviv. The city’s elevation varies between 380 m above sea level in its hilly areas and 5 m above sea level at the foothills. In line with their natural conditions, neighborhoods in the hilly areas typically include cul-de-sac street layouts, and those in the foot-hills include more compact grid street networks. Rishon LeZion, the fourth largest Israeli city (247.3 thousand inhabitants), is centrally situated 13 km east-south of Tel-Aviv with a flat topography (48 m above sea level on average). The eastern part of the city characterized by dense urban environment and the western part includes newer semi-suburban development. Beer Sheva, the eighth largest Israeli city (205.8 thousand inhabitants), is a southern central city just on the edge of the desert, 115 km south east of Tel Aviv with a flat topography (280 m above sea level on average) and very high temperature levels, especially in the summer. Its form is mostly urban, but it also includes semi-suburban developments, mostly in its outskirts. On the socioeconomic rank of 255 cities/towns where 1 is the lowest SES, Haifa ranks 188, Rishon LeZion ranks 204 and Beer Sheva 133 [41]. Overall, Israeli cities pose an intriguing setting for studying obesogenic environments. In spite of their overall high densities, their street patterns vary from compact grid streets in the cities centers to disconnected loops and cul-de-sacs in the semi-suburban areas. These development patterns are not the traditional city-suburb divide that is typical to North American and Australian cities, but rather may be more relevant for European, Asian and Latin America urban settings. These nuances are important given the notion the environmental influences on health are known to vary by context. The study was approved by the University of Haifa and the Maccabi health services ethics committees.

### 2.2. Telephone Survey

A telephone survey was conducted by the University of Haifa Statistics Consulting Unit during February–March of 2015. Records were received from Maccabi Health Services (MHS) and included 10,720 adolescents randomly allocated from these three cities, 1034 were removed from the sample due to: more than one sibling from the same household, telephone number missing, duplications of households or wrong addresses. The records included the following details: ID, full name, date of birth, sex, address, contact information, height, weight, BMI, dates of measuring and status in weight scale (underweight, overweight, obese). 

An initial call was made to potential participants’ parents in order to receive approval for interviewing their child. Parents were informed regarding the procedure of the interview and the questionnaire. Screening was conducted for inappropriate age, adolescents who stay in different houses and adolescents who are physically incapable of performing PA. After receiving parents’ permission, participants were contacted and were asked to participate. The time between calling parents and calling adolescents varied. In some cases, the adolescents answered right after receiving the parent permission, and in others, adolescents were not available and an additional call was made later in the day or during the next few days. Those who gave consent were then interviewed. Each interview lasted between 15 to 20 min. In total, 3059 numbers were attempted and 904 full interviews were conducted. There were 1383 refusals. The total response rate was 29.5% and the net response rate was 45.2% (for a full description of the response rate see Supplementary Table S1). Throughout the survey sampling was balanced for age and sex across the three cities.

The survey questionnaire was built for the study purposes and was based on questionnaires from the literature and previous national surveys about PA and nutrition among adolescents [42,43,44,45,46]. It included four main parts: (1) PA, (2) sedentary behaviors, (3) nutritional home environment and (4) socio-demographic details. The raw BMI data from the MHS records was adjusted based on the 2007 WHO’s BMI-for-age growth reference for boys and girls [47,48] and the build environment data was extracted using Geographic Information System (GIS) based on respondents’ addresses.

The survey questionnaire was pilot tested in a telephone survey among a sample of 20 adolescents using test-retest protocol for reliability. Participants in the pilot study were sampled from the general population using convenience and snowball sampling. All of the pilot study findings were analyzed and items with low correlations (<0.3) were removed or altered. Only strong items that were highly correlated in the baseline and follow-up surveys were included in the final questionnaire. For example for the item: “How many times per week do you walk in the park/playground/sports field?” the correlation was r = 0.76 (*p* = 0.04); for the item: “How many times per week are you walking or running in the neighborhood for fun or for sports?” the correlation was r = 0.71 (*p* = 0.001); and for the item: “In my house there are always fruits or vegetables that I can take and eat," the correlation was r = 0.75 (*p* < 0.001). The questionnaire was also tested for face, content and consensual validity using experts from the field of health promotion, health environments and nutrition, who examined it and gave feedback on the items included. 

### 2.3. Variables

Variables for this study were collected from three different sources: the dependent variable—adolescents’ overweight and obesity—was assessed through BMI records from MHS that were adjusted by age and sex. The independent variables included self-reported survey measures (PA, sedentary behavior, nutritional home environment) and were collected from the survey interviews and the objective GIS-based measures (residential density, intersection density, land-use mix and walkability) were calculated from the addresses of the adolescents that we received from MHS. The section below describes the study variables by their data collection method.

The dependent variable—adolescents BMI: Adolescents’ overweight and obesity—was assessed by BMI data extracted from MHS records and adjusted by age and sex. The adjusted scores were divided into two groups according to percentiles: 1. Normal weight (84 percentile and below) 2. Overweight/Obesity (85 percentile and above). These cut-off points were based on the 2007 WHO’s BMI-for-age growth references for boys and girls [47,48]. 

### 2.4. Independent Variables

Self-reported data including: (1) PA behaviors based on a summary score of five items regarding frequency of walking or doing PA on a scale of 1–4 (1 = never, 2 = less than two weeks, 3 = once-twice times a week, 4 = more than 3 times a week). Items included: 1.1. Walking to a park/playground/sports field; 1.2. Walking to a store/shopping center/mall/coffee house; 1.3. Walking to a community center or a youth center; 1.4. Walking or running in the neighborhood for fun or for sports; 1.5. Doing PA in an open public space like a park, playground, sport field, the beach or a small forest. (2) Sedentary behaviors based on a mean score of two questions on a scale of 1–5 (1 = never,2 = less than 1 h a day, 3 = 1–2 h a day, 4 = 2–3 h a day, 5 = more than 4 h a day). Items included: 2.1. In a normal day, how many hours do you sit in front of a television screen?; 2.2. In a normal day, how many hours do you sit in front of a computer/tablet/phone screen? (3) Nutritional home environment based on a mean score of five items on a dichotomy scale of 1 = agree 2 = not agree. Items included: 3.1. In my house there are always fruits or vegetables that I can take and eat; 3.2. In my house there is a cupboard full of candy and salty snacks; 3.3. In my house we eat fresh vegetables or a salad every day; 3.4. Our family eats out or gets take away food at least twice a week; 3.5. In my house we eat most of the meals together with my parents on most days. All items were recorded so that 0 = unhealthy habit 1 = health habit. The recoded scores were divided in to two groups: 0–0.7 = less healthy nutritional home environment, 0.71–1 = healthier nutritional home environment.

### 2.5. GIS-Based Built Environment Variables

The independent variables included the built environment data extracted from existing GIS databases and maps based on respondents’ neighborhoods. Data was obtained from The City of Haifa, The City of Rishon LeZion, The City of Beer Sheva, and The Survey of Israel. It included zoning layers, building layers, road network layers, and a statistical zone layer with total population count. 

The addresses of survey respondents were not available to the researchers to ensure confidentiality. Instead, within-cities statistical areas were used for this analysis, with each participant being assigned to the statistical area where his/her home is located. Statistical areas are small homogeneous geographical areas (3000 residents on average) that reflect the unique characteristics of a certain area inside a city. Statistical areas are determined by Israel’s Central Bureau of Statistics and are parallel to the US Census Block Groups. The area for calculation included an additional 100-m buffer around the statistical areas. Built environment variables were calculated using ArcGIS 10.3 software based on existing GIS protocols for research in the field [49].

The built environment variables included: 1. Residential density: ratio of housing units to the land area devoted to residential use; 2. Land use mix: entropy measures showing evenness of distribution of land area diversity: residential, commercial, and office development, with normalized scores ranging from 0 for single use to 1 for equal prevalence of multiple uses; 3. Street network connectivity (intersection density): number of intersections per square kilometer; 4. A general walkability score that included all of these components was also generated. All of the built environment variables were based on z-scores. The z-scores were separately calculated for each city. The walkability score was calculated using the following formula: Walkability = ZResidential Density + ZEntropy + ZIntersection Density. 

### 2.6. Control Variables

The potential confounding variables included: gender (male/female), and age (grade number—9th, 10th, 11th, 12th). Area SES was obtained from the Central Bureau of Statistics (CBS) classification which provides a code for each adolescent’s place of residence. The CBS classification takes into account 21 parameters representing measures for demography, education, standard of living and receipt of National Insurance (Social Security) welfare benefits, as recorded in the most recent population census conducted in 2006 [50].

### 2.7. Statistical Analysis

Standard univariate analyses were conducted to describe the characteristics of the study population by city. In order to assess the differences in obesity between the three cities, the associations between BMI and the other variables expected to be associated with obesity were analyzed using chi-square and *t*-test according to the type of variable. To identify the association between the built environment measures and BMI adjusted for area SES, gender, PA, sedentary behavior and nutritional home environment, we ran a multivariable logistic model for each city. The groups examined were adolescents with normal weight vs. adolescents with overweight and obesity. The reference group was the overweight and obesity group. All analyses were performed using SPSS Software, version 21 (SPSS Inc., Chicago, IL, USA).

## 3. Results

This study included 904 interviews with adolescents living in three cities in Israel. Table 1 presents the study population by city. In Beer Sheva there were more overweight and obese adolescents, compared to the other two cities (29.2% in Beer Sheva vs. 22.5% and 23.9% in Haifa and Rishon LeZion), but the difference was not significant (*p* = 0.15). The area SES the adolescents resided in was significantly highest for Haifa and lowest for Beer Sheva. Significant differences were found between cities in intersection and resident density and in land-use mix (*p* < 0.0001). Beer Sheva had the highest residential and intersection density and Rishon LeZion had the highest land use mix. Adolescents reported similar levels of PA and sedentary behaviors in the three cities. However, there was a significant difference in the nutritional home environment, in Haifa, a more positive (healthier) environment was reported compared to the other two cities.

Table 2 presents the overweight and obesity rates in each city according to gender, age and nutritional home environment. The major difference between the cities was evident when analyzing by gender. Boys, in all three cities presented higher rates of overweight and obesity, in Beer Sheva this difference was not significant. In Haifa for example, 26.3% of boys were overweight and obese and only 17.9% of girls were overweight and obese, similar to Rishon LeZion. In Beer Sheva however, the levels of overweight and obesity were much higher among girls compared to the other cities (27.2% compared to 18.5% and 19.1%). 

Table 3 presents the association between BMI (normal weight vs. overweight and obesity), area SES, and the behavioral and environmental measures, using bivariate logistic regression models. Area SES was significantly associated with BMI only in Rishon LeZion, where the adolescents with overweight and obesity lived in higher SES areas. In Haifa the association was in the opposite way but did not reach significance (*p* = 0.097). We found no significant association between BMI and objective built environment attributes (walkability, residential density) and self-reported behavior (PA and sedentary behavior) in the total sample, and in Haifa and Rishon LeZion. However, in Beer Sheva, lower levels of walkability and land use mix were associated significantly with higher odds of adolescents being overweight and obese. Higher levels of sedentary behavior were significantly associated with higher odds of adolescents being overweight and obese in the total sample but not when the sample was divided into the three cities.

In order to predict adolescents’ BMI (normal weight vs. overweight and obesity) according to the built environment measures, we used a multivariable logistic regression analysis for each city and controlled for area SES, gender, PA, sedentary behavior and nutritional home environment (Table 4). Each of the built environment variables was added separately to a model due to co-linearity between the built environment measures. 

In Beer Sheva, walkability, intersection density and land use mix were associated with BMI over and above the other variables: Healthy BMI was associated with higher levels of walkability (OR = 0.72, CI: 0.56, 0.92), intersection density (OR = 0.75, CI: 0.56, 1.00) and land use mix (OR = 0.73, CI: 0.55, 0.96). Resident density was not associated with BMI. However, none of the built environment variables in this study explained the BMI in Haifa and Rishon LeZion.

In these multivariable logistic regressions, gender was near significance (*p* = 0.08) in Rishon LeZion and sedentary behavior was near significance (*p* = 0.09) in Haifa (values not presented), suggesting boys had a higher chance of being overweight and obese in Rishon LeZion and the sedentary had a higher chance of being overweight and obese in Haifa. 

## 4. Discussion

The current study examined the associations between adolescents’ overweight and obesity and objective built environment features and self-reported behaviors. The study was conducted among Jewish adolescents in three Israeli cities representing different geographical regions, SES and peripherality: Haifa (high SES peripheral city in the north), Rishon LeZion (high SES central city) and Beer Sheva (low SES peripheral city in the south). The highest level of adolescents’ overweight and obesity was in Beer Sheva. No differences were found between the cities in terms of built environment characteristics and PA and sedentary behaviors. In Haifa, a more positive nutritional home environment was reported. Boys, in all three cities presented higher rates of overweight and obesity. The relationships between the built environment, areal SES, BMI and reported PA and sedentary behaviors were different between the three cities. A higher level of sedentary behavior was significantly associated with overweight and obesity in the total sample. After adjusting for area SES and behavioral factors, we found that the built environment was associated with obesity and overweight only in Beer Sheva, so that overweight and obese adolescents were living in less walkable neighborhoods compared to adolescents with normal weight. 

The current findings show that the association between adolescents’ obesity and the built environment was not consistent across the three cities. Previous studies that examined this relationship have shown contradicting evidence [3,33,34]. All this suggests that the relationship between obesity and the built environment is not direct but rather influenced and mitigated by different individual, social and environmental factors [34]. 

Although in our study we did not find a consistent relationship between obesity and the built environment across the three cities measured, in one of the cities- Beer Sheva, there was an association between BMI and built environment features. Only in this city, the overweight and obese adolescents were living in less walkable neighborhoods compared to adolescents with normal weight. Beer Sheva is the most peripheral, lowest SES, and the smallest city in this study (205.8 thousand residents) [41]. This finding amplifies the role of area SES and possibly other individual and social factors, in the relationship between the built environment and obesity. These findings also support previous evidence of associations between peripherality, lower area SES and lower walkability [38,51]. Previous studies suggested that the benefits of the built environment might be experienced mostly by people who already enjoy health advantages [52,53]. It could be that in wealthier cities such as Rishon LeZion and Haifa, the adolescents depended less on active transport and are anyway less overweight and obese, therefore the built environment does not exert its effect and no association is observed.

The risk of obesity is known to be more prevalent among low-SES populations and areas [54,55]. However, our findings were not completely in agreement with previous studies. Only in one city (Haifa), high area SES was associated with lower BMI but it did not reach statistical significance. In another city (Rishon LeZion), overweight and obesity were associated with higher levels of area SES. In addition, area SES was negatively associated with the built environment measures in Haifa and in Beer Sheva, suggesting that higher SES areas had lower walkability. It should be noted that in Beer Sheva these associations might reflect residential preferences (affluent preferring low-walkable suburban neighborhoods); however, in Haifa, these associations are likely to merely reflect the topography, as higher SES neighborhoods tend to be located up on the hill where streets are shaped as cul-de-sac due to the topography, thereby yielding low walkability. Only in Rishon LeZion, walkability was positively associated with area SES, showing higher built environment walkability in affluent areas. 

These inconsistencies in the relationships between area SES, the built environment and obesity may be explained by the use of area-SES in this study instead of family income, which may have had an influence on the results. Participants from high-income families may have resided in areas that are considered to be low SES areas and vice versa. These inconsistencies between the cities may also indicate that more global, city-level factors such as urban policy and socio-cultural climate, may influence the nature of the relationships between SES, BMI, health behaviors and the built environment, defining the differences between the cities.

In line with previous studies [12,13], BMI was found to be associated with higher levels of sedentary behaviors. Sedentary behaviors were also found to be correlated with higher area SES in Beer Sheva but with lower area SES in Haifa. This finding may indicate different social norms in Beer Sheva that are more similar to traditional developing societies where childhood overweight and obesity is associated with higher area SES levels [38,56]. 

Our findings regarding gender differences may also have important implications for understanding health among adolescents. Boys, in all three cities, presented higher rates of overweight and obesity. These gender differences in BMI were also found in a national study conducted among adolescents in Israel [57] and in previous studies among Israeli children [58]. Literature shows that compared to girls, boys have a higher risk of being obese due to physiological differences [59,60]. Although, boys tend to be more physically active than girls during adolescence [61], several studies have shown that adolescent girls have a more nutritional healthy diet, compared to boys who have higher rates of eating sugary and high fat foods [7,62]. In an international study among 44 countries, boys had higher rates of overweight and obesity compared to girls, in all of the countries examined (including Israel) [7]. Apart from the physiological explanation, there are also social differences between boys and girls, such as weight awareness and dieting, which are more common among girls [7,63].

### Study’s Strengths and Weaknesses 

The sampling was conducted by a list that was extracted from one healthcare provider in Israel. Therefore, the participants were limited to a certain healthcare provider. However, this is less likely to have affected our results as this healthcare service does serve 25% of the Israeli population (36.5% in Haifa, 24.3% in Beer Sheva and 41.9% in Rishon LeZion) [64]. In addition, we used a sample from three different cities and geographical areas to represent the target population and eliminate sampling bias. Since this study included a questionnaire that examined health behaviors, the variables could be subject to information and social desirability bias. Since the study was conducted among mostly Jewish populated cities, we did not include Arab adolescents. Therefore, our findings could not be generalized to both populations, but rather only to Jewish adolescents. On the other hand, limiting our sample to Jewish adolescents likely eliminated potential confounding effects that may have occurred and that were observed in prior research.

Studies that examined the correlations between objective and self-reported PA found high correlations between the two types of measurement [65], although indicating the advantage of the objective measures over self-reported PA [66]. However, we used objective measures like BMI and GIS variables that were not subjected to participants’ responses. In addition, using a telephone survey made it easier to produce a representative sample in a short time period and with an appropriate response rate. The anonymity of a telephone survey allowed the participants to feel comfortable answering personal questions about their health and lifestyle [67].

Due to privacy reasons, Macabbi did not provide us the participants’ addresses. Therefore, all the spatial variables were based on the broader sub-neighborhood statistical zones. This weakens the validity of the built environment factors to represent the respondents, as previously acknowledged through the modifiable areal unit [68]. The walkability index was calculated based on zoning maps rather than land use maps and the zoning map for Beer Sheva was missing and speculated by us based on building zoning map. However, there is no reason to believe that the size of the bias was differentially distributed between the variables in any of the dependent-independent variable associations examined. At most, there may be an attenuation of the associations detected. The potential confounding of the association between dependent-independent variables was addressed at the analysis stage, using multivariate analysis.

## 5. Conclusions

To conclude, this study examined the relationship between adolescents’ BMI and the built environment and self-reported health behaviors in a diverse sample, including three cities representing different geographical regions, SES and natural conditions. The relationships between the built environment, PA and BMI, manifested differently in each city, with Beer Sheva demonstrating different patters compared to the other two cities. It seems that in the low SES city, the built environment may be associated with overweight and obesity. These findings indicate that walkability and BMI may be associated only in specific circumstances and therefore changes in the built environment alone may not help in tackling the obesity epidemic. Other factors may need to be taken into account. The differences between the cities suggest that efforts to improve the built environment should also be based on city-level factors such as urban policy and socio-cultural climate rather than intra-city level factors like walkability and nutritional environment. Taken together, it may be recommended that future interventions to prevent adolescent obesity will use environmental strategies to improve walkability, especially in disadvantaged peripheral areas. Promoting walking and using active transportation was found previously to reduce BMI [69]. Such interventions may not only prevent adolescent’s obesity in those areas, but rather also may help in reducing spatial socioeconomic disparities in adolescent’s obesity.

## Figures and Tables

**Table 1:** Sample characteristics by city, mean, standard deviation for continues variables, and percent for categorical variables, *p* values for difference between cities, *n* = 904.

Variable	Category	Haifa *n* = 299		Rishon LeZion *n* = 305		Beer Sheva *n* = 300		*p*
		Mean, SD	*N* (%)	Mean, SD	*N* (%)	Mean, (SD)	*N* (%)	
BMI	Normal		207 (77.5)		204 (76.1)		206 (70.8)	0.15
	Overweight and Obese		60 (22.5)		64 (23.9)		85 (29.2)	
Age	9th grade		12 (4.0)		10 (3.3)		14 (4.7)	0.5
	10th grade		101 (33.9)		111 (36.6)		94 (31.4)	
	11th grade		115 (38.6)		96 (31.7)		111 (37.1)	
	12th grade		70 (23.5)		86 (28.4)		80 (26.8)	
Gender	Boys		150 (50.2)		155 (50.8)		147 (49.0)	0.9
	Girls		149 (49.8)		150 (49.2)		153 (51.0)	
SES		15.68 (3.67)		13.82 (2.3)		11.07 (3.6)		**<0.0001**
PA sum		11.10 (3.17)		11.25 (3.43)		11.28 (3.4)		0.78
Sedentary behavior mean		3.41 (0.87)		3.49 (0.87)		3.53(0.97)		0.28
Nutritional home environment mean	Less healthy environment (0–0.7)		144 (44.3)		135 (44.3)		144 (44.3)	**0.001**
	Healthy environment (0.71–1)		167 (55.7)		170 (55.7)		167 (55.7)	
Walkability		0.09 (0.73)		−0.01 (1.05)		−0.08 (1.16)		0.12
Intersection density		−0.68 (0.69)		0.03 (0.85)		0.65 (0.98)		***p* < 0.0001**
Resident density		−0.39 (0.82)		0.12 (1.01)		0.26 (1.03)		***p* < 0.0001**
Land-use mix		−0.33 (0.90)		0.48 (0.84)		−0.16 (1.05)		***p* < 0.0001**

Bold numbers represent significant differences between cities.

**Table 2:** Percent of obese and overweight adolescents according to city, age, gender and nutritional home environment (Chi-square).

Variable	Category	Haifa *n* = 299	Rishon LeZion *n* = 305	Beer Sheva *n* = 300	Total
		*N* (%)	*N* (%)	*N* (%)	*N* (%)
Age	9th grade	1 (11.1)	2 (33.3)	4 (30.8)	7 (25.0)
	10th grade	17 (19.3)	20 (21.5)	27 (31.4)	64 (24.0)
	11th grade	24 (22.2)	25 (29.8)	33 (29.7)	82 (27.1)
	12th grade	18 (29.5)	16 (19.3)	21 (26.3)	55 (24.6)
	*p*	0.41	0.37	0.90	0.84
Gender	Boys	36 (26.3)	39 (28.5)	45 (31.3)	120 (28.7)
	Girls	24 (18.5)	25 (19.1)	40 (27.2)	89 (21.8)
	*p*	**0.05**	**0.048**	0.26	**0.009**
Nutritional home environment	Less healthy environment (0–0.7)	18 (20.5)	27 (23.3)	41 (31.8)	86 (25.8)
	Healthy environment (0.71–1)	42 (23.5)	37 (24.3)	44 (27.2)	123 (24.9)
	*p*	0.35	0.48	0.23	**0.42**

Bold numbers represent significant differences in overweight and obesity in each category.

**Table 3:** Adjusted bivariate regression models predicting adolescents’ Body Mass Index (BMI) by socioeconomic status (SES), the built environment and physical activity (PA).

Variable	Haifa *n* = 267		Rishon Lezion *n* = 268		Beer Sheva *n* = 291		Total	
	OR	CI	OR	CI	OR	CI	OR	CI
Areal SES	0.94	0.87–1.01	1.19 *	**1.03–1.38**	1.02	0.95–1.09	0.98	0.94–1.03
Z-Walkability	1.34	0.93–1.95	1.19	0.91–1.56	0.74 *	**0.59–0.93**	0.95	0.81–1.11
Z-Intersection density	1.15	0.78–1.71	0.92	0.66–1.30	0.80	0.62–1.04	1.00	0.86–1.17
Z-Residential density	1.36	0.97–1.90	1.06	0.80–1.40	0.89	0.70–1.14	1.07	0.92–1.25
Z-Land-use mix	1.05	0.77–1.44	1.11	0.79–1.55	0.76 *	**0.60–0.98**	0.92	0.78–1.07
PA	1.09	0.99–1.18	1.07	0.99–1.17	0.98	0.91–1.05	1.04	0.99–1.09
Sedentary behavior	1.333	0.95–1.86	1.09	0.78–1.51	1.25	0.96–1.63	1.23 *	**1.03–1.47**

* *p* < 0.05. Bold numbers represent significant ORs.

**Table 4:** Multivariable logistic regression models to predict adolescents’ BMI in each city by the built environment measures and controlled for area SES, gender, PA, sedentary behavior and nutritional home environment.

Variable	Haifa *n* = 263			Rishon LeZion *n* = 254			Beer Sheva *n* = 276		
	OR	*p*	CI	OR	*p*	CI	OR	*p*	CI
Z-Walkability	1.31	0.23	0.84–2.04	1.08	0.63	0.80–1.44	**0.72**	0.009	0.56–0.92
Z-Intersection density	1.07	0.79	0.65–1.78	0.91	0.62	0.63–1.32	**0.75**	0.05	0.56–1.00
Z-Residential density	1.25	0.33	0.80–1.94	1.03	0.83	0.77–1.39	0.89	0.40	0.67–1.17
Z-Land–use mix	0.97	0.85	0.68–1.37	1.13	0.49	0.80–1.60	**0.73**	0.026	0.55–0.96

Bold numbers represent significant ORs.

## Supplementary Materials

The following are available online at https://www.mdpi.com/1660-4601/16/9/1579/s1, Table S1: Survey’s response rate description.
