# Supplementary material for: The Relationships between Adolescents’ Obesity and the Built Environment: Are They City Dependent?
Source: Int J Environ Res Public Health. 2019 May 6;16(9):1579. doi: 10.3390/ijerph16091579 (PMC6539234; doi:10.3390/ijerph16091579)
Supplement: Supplementary file 1 [file ijerph-16-01579-s001.pdf]

Supplement 1. Survey's response rate description

Classification	n
1. Completed interview	**904**
2. Partial interview	**20**
3. Screening 1: no child between 15-18	**67**
4. Screening 2: Address is not included in the study	**12**
5. Screening 3: child does not live at household full time	**96**
6. Screening 4: child has a physical disability	**61**
7. Screening 5: parent's refusal	**288**
8. Child could not be contacted	**341**
9. Child refusal	**243**
10. Communication ceased (after multiple rejections)	**44**
11. Respondents' difficulties answering questions (language barriers)	**30**
12. No cooperation by the respondent	**491**
13. Rejects (were still in process at the end of survey)	**439**
14. Other cases where it was not possible to conduct the interview	**21**
Total numbers attempted	**3057**

	n	Response rate
Total refusals	1383	**45.2%**
General total	904	**29.5%**
